# A new diagnostic model to understand the root causes of failing waste collection in developing countries, with example applications in Latin America

**DOI:** 10.1177/0734242X251362590

**Published:** 2025-09-28

**Authors:** Hans Breukelman, Harold Krikke, Ansje Löhr

**Affiliations:** 1 Faculty of Management, Science and Technology, Open University of the Netherlands, Heerlen, the Netherlands; 2 Department of Environmental Sciences, Faculty of Science, Open University of the Netherlands, Heerlen, the Netherlands

**Keywords:** Waste collection, service coverage, developing countries, system dynamics, root causes, Latin America

## Abstract

Developing countries around the world are challenged to provide their citizens with access to waste collection. But performances are poor and services are mostly limited to the urban areas. So far, little is known about the root causes of this deficient achievement. This Short Communication summarises the results of a research programme that used system dynamics to analyse the problem in a retrospective and prospective manner. The programme has resulted in a scientifically grounded and tested diagnostic model that enables both scientists and practitioners to dig below the symptoms of the problem, to improve understanding of what is actually going on, to make predictions under different scenario’s and to test interventions. The model resulting from this research clearly shows how two clusters of societal processes compete. One cluster holds the processes on economy, quality of government, national revenues and available budgets. The other cluster comprises the processes that use these budgets. For the Latin American countries, lack of money is not the key cause. The problem must be sought in the close interaction between quality of government and the efficiency of collection services. Specific interventions are suggested for the case of Bolivia.

## Introduction

Developing countries struggle to provide waste collection services to all of their citizens. Research shows that currently, around 35% of the global population is not being serviced with waste collection (Lenkiewicz, 2024). Almost all of these 2.8billion people live in lower- or middle-income countries, and there, mainly in the rural areas (Lenkiewicz, 2024). This absence of waste collection has direct negative effects on these people’s health and it worsens their living conditions. In addition, it strongly increases the global emissions of greenhouse gases (GHGs) and the discharge of waste plastics into the environment (Ferronato and Torretta, 2019; Kaza et al., 2018; MacAfee and Löhr, 2024; Wilson, 2023).

The success of governments in providing their citizens with proper waste collection is the outcome of many interwoven societal processes. Knowing more about these processes can be helpful in designing interventions that may improve the health and well-being of the poorest people in these countries, and meanwhile give support to policies on abating GHG emissions and marine litter. A literature review on the current status of research in this field describes the need for holistic diagnostic tools (Breukelman et al., 2019). The study showed the inability of the professional field to dig below the surface of symptomatic observations such as a lack of money, knowledge, equipment, regulations, policies and enforcement. The most recent report of UNEP showed that there is still no progress (Lenkiewicz, 2024). Apparently, the problem is complex in the sense that there are many intertwined societal processes involved and because these processes are far from static or stable. The UNEP report referred to concludes that this situation has all the characteristics of a wicked problem (Lenkiewicz, 2024). Over the last 10–20 years, the professional field has attempted to dig deeper, but the methods that were developed did not surpass the level of benchmarks and checklists (Breukelman et al., 2019; Wilson et al., 2014). It is concluded that systems thinking may enhance our understanding of the underlying societal processes for failing waste collection. It may keep us from speculations on the causes and from ineffective, or even counterproductive, interventions (Breukelman et al., 2019).

This communication describes a research programme that used the full width of system dynamics modelling (SDM) in order to design a diagnostic model that can be used to pin down the root causes for failing waste collection in developing countries (Breukelman, 2025). The research had a focus on Latin America and culminated in a case study for Bolivia. Nevertheless, the model is applicable in developing countries around the world.

## Methods and results

### System dynamics modelling

**Figure 1.**
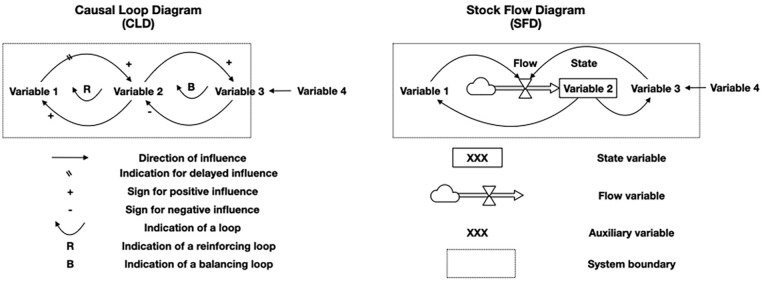
Explanatory examples for a CLD and an SFD, including the used notation (for further guidelines see Morecroft, 2015; Sterman, 2000). CLD: causal loop diagram; SFD: stock flow diagram.

SDM was developed almost 60 years ago by Jay Forrester as a method to model complex and dynamic systems. It uses qualitative and quantitative modelling to describe the most important variables in a system, including the ways these variables influence each other, how these influences change with time and how they affect the system as a whole and, in particular, the target variable of concern (Forrester, 1969; Forrester and Senge, 1979; Morecroft, 2015; Sterman, 2000). In the research described in this article, the target variable was defined as the percentage of the total population that is serviced with regular waste collection services. Figure 1 gives a simple example of an SD model and a description of the notations that are used.

The first step in SDM is often qualitative modelling, and the product is called a causal loop diagram (CLD). A CLD is a theoretical proposition about the system. It depicts the most important variables of a system and connects these variables through arrows when there is a relation between them. The arrows in a CLD give qualitative descriptions of the relations. They show the causal direction of the influence, its polarity (is the effect positive or negative) and whether there is any delay in this influence. Relations can form loops that may be either balancing (caused by an uneven number of negative arrows) or reinforcing (caused by an even number of negative arrows). A CLD can already play an important role in mapping and explaining the behaviour of a system.

The second step is one of quantitative modelling in which the descriptive relations of a CLD are translated into mathematical equations. The result is a so-called stock and flow diagram (SFD) that is fit for empirical research. It distinguishes between stock, flow and auxiliary variables. Stock variables represent the state of a system; they grow or diminish when there is a flow coming in or going out. They are like the water level in a bathtub, keeping their value when all incoming and outgoing flows stop. An example is the population size of a country at a given moment. Flow variables would then be the number of births, deaths and migrations per year. Auxiliary variables are intermediate variables that are helpful in describing the system. The mathematical equations, describing the relations, make use of constants and parameters. The equations, constants and parameters can be based on literature, estimates, expert views or on any other source.

It is important to distinguish between endogenous and exogenous variables. Endogenous variables are those that are included in the system and that have at least one incoming relation with other variables in the system. Exogenous variables are excluded from the system itself but can still have their influence on variables in the system. They are not (or only very weakly) affected by any variables within the system. This makes drawing the boundaries of a system an important part of modelling the system as it excludes those variables that experience no (or minimal) influence from the system itself.

### Qualitative modelling

**Figure 2.**
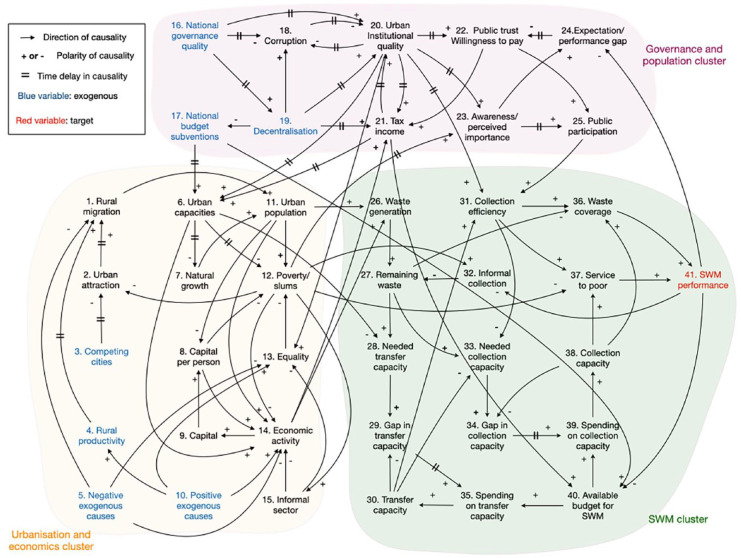
Causal loop diagram integrating all variables and relations with regard to governance (purple), urbanisation (yellow) and waste management (green). Used notation as indicated in this figure and in Figure 1.

The qualitative part of the study was published in 2022 (Breukelman et al., 2022). It used literature and expert views to identify the most important variables and relations, and they were brought together in the CLD as given in Figure 2. Another qualitative method, based on graph theory, was then used to characterise the variables, their relations, the branched causal subsystems and the feedback loops in this CLD. The results revealed a first general conclusion being that the root cause of the problem must be sought somewhere in the interplay of strong population growth, urbanisation and economic growth. This concurrence can lead to mutually reinforced dynamics amongst the variables. The effect may be so strong that it cannot be managed by governments, not even in situations where these governments are able to improve their own quality and to generate needed budgets. But the reinforcement can also go downwards, pushing countries into a vicious cycle of deteriorating economic growth, state revenues and quality of government, generally referred to as the poverty trap.

### Quantitative modelling

Quantitative modelling was used for understanding the actual strength of the societal processes and the way they influence the behaviour of the system as a whole. This part of the research was published in 2024 (Breukelman et al., 2024). The relations of the CLD were turned into the mathematical format of an SFD. Literature, expert views, estimates and assumptions were used to set up the model’s initial formulas and the values of its parameters. Vensim software was used to construct the SFD and to produce simulations of the behaviour of the system over time (Vensim-Systems, n.d.).

The next step was to test this general model against historical real-life datasets and to calibrate the model’s parameters in order to produce improved simulations. This calibration was done with the same software and with historical country datasets that were found for six individual Latin American countries (Belize, Bolivia, the Dominican Republic, Ecuador, Panama and Paraguay). After calibration of the parameters per country, the model produced good fits with the historical datasets for almost all variables and for all six countries. The resulting parameter sets per country were then used to compare the countries. This comparison shows similarities and differences. As already shown in the qualitative model, population growth shows a strong and dual effect on the budgets for waste collection: one of increasing budgets and one of diluting them. None of the countries seems to be in the poverty trap. All six countries show economic growth, but still the differences are remarkable. For Belize, Ecuador and Paraguay, the available government budget per inhabitant shows growth, but for Bolivia, the Dominican Republic and Panama, it does not. Speed of urbanisation was expected to play a strong negative role as it may overwhelm authorities, but it only seems to be a problem in Bolivia. There, the cities appear to be unable to absorb the influx of new inhabitants. For all countries, growth in Gross Domestic Product (GDP) shows strong effects on government revenues and governance quality because the variables are in an important reinforcing loop that directly affects the availability of waste collection services. With the exception of Bolivia, the citizens’ willingness to participate in waste collection does not seem to play an important role. All countries show that a small growth in the number of citizens serviced with waste collection needs a more than proportional growth in needed budgets. The explanation is that logistical processes become more difficult and expensive when trying to reach citizens in more remote outskirts and in the rural areas. Paraguay and Ecuador perform relatively well. There, quality of governance is slowly but steadily improving and, despite their low government revenues, they are able to show growing percentages of serviced citizens, overcoming the dilution effect of growing populations. Belize shows an opposite, negative development. And finally, the Dominican Republic and Panama show a moderate-to-strong inertia across almost all parameters. Apparently, doing well on the economic side does not automatically mirror itself into increased performance on waste collection.

### Case study Bolivia

**Figure 3.**
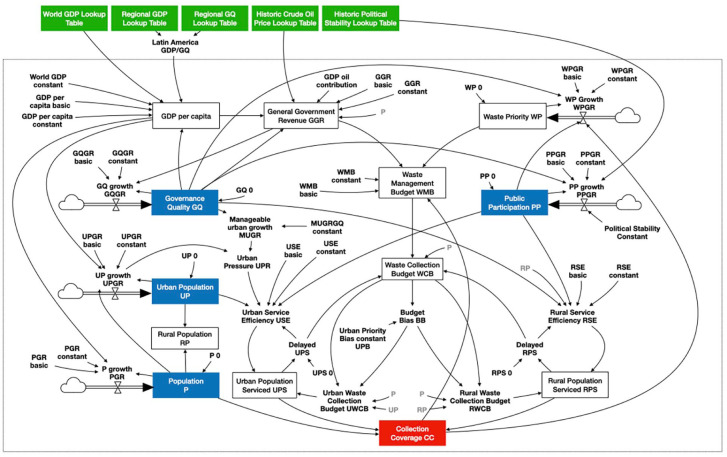
Stock flow diagram as developed for six Latin American countries (exogenous variables in green, stock/state variables in blue and target variable in red). Used notation as indicated in Figure 1.

**Figure 4.**
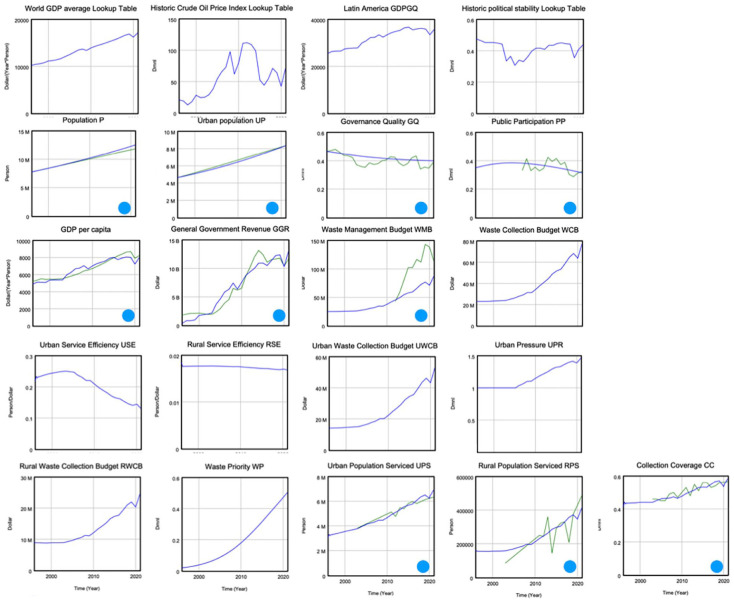
Model simulation for Bolivia for the period 1996–2021. The blue lines represent the historical data, and the green lines (in boxes with blue dot) represent the simulated variables based on the calibrated parameter sets. The calibration aims at producing the best fit between the blue and green lines. The target variable is CC, in the bottom-right box. CC: collection coverage.

The last part of the research focussed on improving the understanding of the role of budget availability for waste collection, of the operational efficiency of these services, of the participation of the public in these budgets and operations and of the more-detailed influence of urbanisation. This was done by using the same model in a more-detailed case study in Bolivia. The SFD was refined and augmented based on interviews with experts in the country. The visual representation of the resulting model is shown in Figure 3. It was then calibrated against additional historical datasets, leading to the resulting simulations shown in Figure 4. The results were presented in the final report of the research (Breukelman, 2025).

The model simulations lead to a concluding narrative for Bolivia, and it goes as follows. Over the last 25 years, the country has been able to increase its governmental revenues at a rate stronger than the growth in population. In the same period, the priority on waste management has also grown and one could have expected that available budgets for waste collection would have taken a growing percentage of government revenues, but that did not happen. Waste collection budgets grew, and even stronger than the population, but it was not enough. The logistical logic of diminishing returns brought down the efficiency of these public services and apparently, this decline was stronger than the rise in budgets. Two other causal processes added to the nosedive of efficiency. The first one is an almost continuous decline in needed public participation in and appreciation of these services, due to low governance quality and years of political unrest. It paralyses the functioning of public departments who have to do the job on a daily basis. The second one is overurbanisation, a rate of urbanisation that cannot be absorbed by the cities. The combined effect of all three processes has brought the increase in service coverage to a standstill, at around 60% of the population.

Using the model for projections and for defining interventions can show what could work in a country and what not. In the case of Bolivia, proceeding with Business-as-Usual would nearly stall further progress on providing a larger percentage of the population with waste collection. The analysis shows a preference for a focus on campaigns that increase the public appreciation of and participation in waste services, decoupled from the (lack of) appreciation of the authorities as a whole. Of course, increasing budgets may be helpful, but their effect will be less then proportional. A focus on efficiency and professionalism in logistics will also be effective but not as strong as increasing public participation.

## Discussion and conclusions

**Figure 5.**
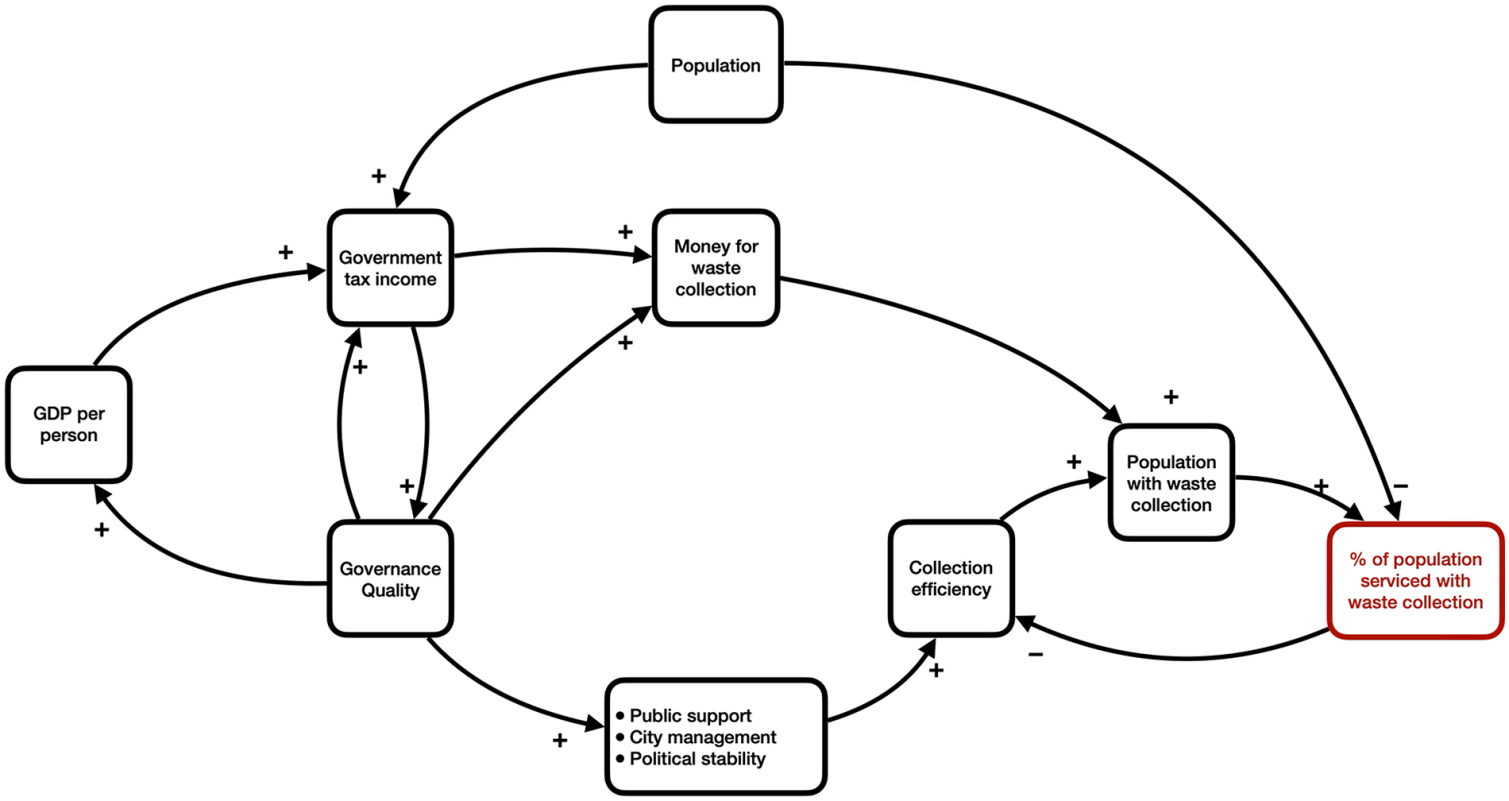
Simplified visual representation of the most influential parts of the system leading to deficient waste collection in the six Latin American countries of this research.

The research shows that SDM is capable of providing a diagnostic tool for finding the causal structures that are at the root of failing waste collection. The resulting SFD (Figure 3) can be used for any country, but it needs the work of experts in order to customise it, to find the right datasets and to perform the calibrations and simulations. For the model to be also useful for other practitioners and policymakers, it needs to be translated into a simplified visual representation that enables a more practical use in presentations and discussions. Integrating the modelling results for the Latin American countries may then lead to the representation of the relevant societal processes as shown in Figure 5. It shows the reinforcing loops of economic growth, governance quality and government revenues on the left-hand side. This part can be looked at as the budget-providing cluster. The reinforcement can be slow or fast, and it can reinforce itself upward and downward. When downward, it represents what development economists call the poverty trap, a vicious circle that is difficult to escape once a country is in it. The right-hand side represents the budget-consuming cluster. It holds the balancing loop of diminishing returns, consisting of the interaction between service efficiency and collection coverage. The left-hand side provides money for the right-hand side. But the two sides are also connected at the bottom through processes on public participation, urban management and political stability. All three are governed by the quality of the government. If this quality is low and/or going down, it deteriorates the efficiency to levels below what could be expected based on regular diminishing returns. Population growth, at the top, has its two-sided effect of increasing and diluting the available budget for collection.

The professional field of waste management may benefit from this research. It now has a diagnostic tool that helps to understand the societal processes that lead to poor waste collection. The model does not negate symptoms such as ‘lack of money, knowledge, laws and equipment’ but allows us to see how this lack is the result of competing or conspiring mechanisms. As could have been expected: the cure is not in standard quick fixes such as more money, better training, new laws or more technology. Using the model can lead to increasing our understanding. It may help us to see what is really needed and to tailor the solutions in such a way that they may influence the more specific underlying mechanisms. But maybe even more important is the fact that understanding these causal mechanisms is a prerequisite to understanding the difficult situation, many governments of developing countries are in.
